# Evaluating usability of and satisfaction with mHealth app in rural and remote areas—Germany GIZ collaboration in Bosnia-Herzegovina to optimize type 1 diabetes care

**DOI:** 10.3389/fdgth.2024.1338857

**Published:** 2024-06-17

**Authors:** Bushra Ali Sherazi, Stephanie Läer, Snijezana Hasanbegovic, Emina Obarcanin

**Affiliations:** ^1^ Institute of Clinical Pharmacy and Pharmacotherapy, Heinrich Heine University, Düsseldorf, Germany; ^2^ Institute of Pharmacy, Faculty of Pharmaceutical and Allied Health Sciences, Lahore College for Women University, Lahore, Pakistan; ^3^ Pediatric Clinic, University Clinical Center Sarajevo, Sarajevo, Bosnia and Herzegovina; ^4^ Lee Kong Chian School of Medicine, Nanyang Technological University Singapore, Singapore, Singapore

**Keywords:** children and adolescents, mobile health applications, type 1 diabetes mellitus, rural and remote areas, specialized diabetes care

## Abstract

**Background:**

Type 1 diabetes mellitus (T1DM) management in children and adolescents requires intensive supervision and monitoring to prevent acute and late diabetes complications and to improve quality of life. Digital health interventions, in particular diabetes mobile health apps (mHealth apps) can facilitate specialized T1DM care in this population. This study evaluated the initial usability of and satisfaction with the m-Health intervention Diabetes: M app, and the ease of use of various app features in supporting T1DM care in rural and remote areas of Bosnia-Herzegovina with limited access to specialized diabetes care.

**Methods:**

This cross-sectional study, performed in February–March 2023, evaluated T1DM pediatric patients who used the Diabetes: M app in a 3-month mHealth-based T1DM management program, along with their parents and healthcare providers (HCPs). All participants completed self-administered online questionnaires at the end of the 3-month period. Data were analyzed by descriptive statistics.

**Results:**

The study population included 50 T1DM patients (children/parents and adolescents) and nine HCPs. The mean ± SD age of the T1DM patients was 14 ± 4.54 years, with 26 (52%) being female. The mean ± SD age of the HCPs was 43.4 ± 7.76 years; all (100%) were women, with a mean ± SD professional experience of 17.8 ± 8.81 years. The app was reported usable in the domains of ease-of-use and satisfaction by the T1DM children/parents (5.82/7.0), T1DM adolescents/young adults (5.68/7.0), and HCPs (5.22/7.0). Various app features, as well as the overall app experience, were rated positively by the participants.

**Conclusion:**

The results strongly support the usability of mHealth-based interventions in T1DM care, especially in overcoming care shortage and improving diabetes management and communications between HCPs and patients. Further studies are needed to compare the effectiveness of apps used to support T1DM management with routine care.

## 1 Introduction

Type 1 diabetes mellitus (T1DM), a common chronic condition in children and adolescents, requires lifelong insulin therapy, regular blood glucose monitoring, diabetes education, and collaborative care to achieve favorable treatment outcomes (1, 2). Personalized holistic care with frequent monitoring and adjustments of insulin doses has been recommended (3), with optimal adherence to the devised treatment plan, along with adequate self-management activities, being the cornerstone of effective T1DM management (4). Suboptimal adherence to insulin regimens, as well as to food and exercise recommendations, is common in pediatric patients with T1DM, resulting in poor glycemic control (5–8), increased morbidity, and premature mortality (9). Suboptimal diabetes control during childhood can lead to the development of late diabetes complications when these patients reach adulthood (5, 10). The costs of diabetes complications are significant and represent a substantial economic burden on healthcare systems worldwide (11–14).

Although the incidence of T1DM in children and adolescents is highly variable, this incidence and the global burden of T1DM is increasing worldwide (1, 15, 16). In 2022, a total of 8.75 million people were living with T1DM worldwide, with 1.9 million living in low- and lower-middle-income countries, including 1.52 million aged <20 years, accounting for 182,000 deaths in 2022 (17). According to the International Diabetes Federation (IDF), the highest number of patients with T1DM in 2022 lived in Europe, followed by North America and the Caribbean regions (17). The highest incidence of T1DM in upper-middle-income countries were in Europe and Brazil (1). The World Health Organization (WHO) has reported that the prevalence of diabetes in Bosnia-Herzegovina (BiH) was 9.3%, being one of the highest rates in Europe (17). The annual incidence of T1DM per 100,000 inhabitants under age 18 years was 7.5 in the peripheral parts of BiH from 1998 to 2010 and 6.5 in the central region of BiH from 1999 to 2004 (18). Unofficial data estimate that the number of newly diagnosed patients has doubled in the last 10 years. The unreported numbers are likely much higher, as BiH does not yet have a nationwide registry for T1DM.

The everyday management of T1DM significantly burdens the affected patients and their families in BiH (19). Healthcare resources are insufficient to cope with the growing demand, with resources in BiH remaining below the regional average for universal health coverage owing to many factors, including the limited-service capacity and poor access among the most disadvantaged members of the population (20). There are only five diabetes reference centers nationwide, with only six specialist pediatric diabetologists, who practice in the major urban areas, Sarajevo, Tuzla, Zenica, Mostar, and Banja Luka (21). BiH, however, remains one of the most rural countries in Europe, with around 60% of the population living in rural areas (22). In rural and remote areas of BiH, children with T1DM are diagnosed and provided with insulin therapy by healthcare centers and ambulances (21). A structured diabetes training provided by an interdisciplinary diabetes team, which is highly essential for controlling the disease according to International Society for Pediatric and Adolescent Diabetes (ISPAD) guidelines (2), is often unavailable. Children with diabetes-related complications are transferred to the closest diabetes reference centers, which are often at full capacity and cannot take on additional patients. The COVID-19 pandemic worsened the situation for these patients. One study from BiH reported that treatment of diabetes complications had a major share in overall diabetes management costs (18).

The American Diabetes Association (ADA) has recommended regular follow-up, ongoing nutrition and diabetes self-management education (DSME) and support (DSMS), and access to specialized healthcare as necessary for effective T1DM management (23). Technology-based interventions can be helpful in patients who miss appointments and/or have poor accessibility to specialized T1DM care (10, 24), as these interventions enable remote monitoring and increase access to evidence-based practices outside conventional clinical settings (25, 26). The use of diabetes technology has increased markedly among children and adolescents with T1DM worldwide (27), with guidelines recommending that it be integrated into pediatric diabetes care (2, 28). The ADA has classified diabetes technology into three categories: hardware, devices, and software, which help patients manage their diabetes (29). mHealth interventions have been recommended for improving diabetes management among young people (30, 31). One such example is the increasing use of diabetes mobile apps to support disease management, prevent diabetes-related complications, and improve overall quality of life (32, 33). Diabetes apps have been reported effective in improving clinical outcomes and diabetes self-management (31, 34–37). The functions of diabetes apps developed to date have been found to vary, as various apps have focused on blood glucose monitoring, self-management, motivation for medication adherence, and lifestyle modifications (38, 39). Tailored education, timely feedback (26), remote monitoring, and follow-up (39) can enable diabetes apps to reduce the risks of increased HbA1c levels, psychosocial problems, and the development of complications associated with disrupted clinic visits (30). Moreover, diabetes apps have great potential for T1DM management in children and adolescents owing to the widespread, viability, and acceptance of the use of technology among young persons (24, 40, 41).

The development of diabetes apps and evidence supporting their efficacy and effectiveness in T1DM management (24, 40, 42–45) suggest that the use of these apps can effectively extend patient-centered care to remote and rural areas of BiH with limited access to specialized diabetes care, as well as providing frequent points of contact with specialist pediatric diabetologists. Evaluations of mHealth interventions in different contexts can influence their implementation, such as the settings in which they are used, the HCPs, and the entire implementation process (46). Accordingly, the current study was designed to assess the initial usability of a mHealth intervention among children and adolescents with T1DM, as well as their parents and HCPs, after using the Diabetes: M app for three months. User satisfaction, experience, and the perceived usefulness of various mHealth app features were investigated using a questionnaire. Findings of this study may provide reference points for the usability of the mHealth app in T1DM management from the perspectives of pediatric patients, their parents, and HCPs in underserved and remote areas.

## 2 Materials and methods

### 2.1 Study design and data collection

This cross-sectional survey conducted in February–March 2023 collected data related to user satisfaction, experience, and the usefulness of different features of the Diabetes: M app in managing T1DM. Pediatric patients with T1DM, along with their parents and HCPs, who had been enrolled in the 3-month mHealth-based T1DM management program, trained and connected through a digital health network, were invited to participate in the present survey. This T1DM management program established a digital network that included all children, adolescents, and young adults with T1DM across Middle Bosnia Canton in Bosnia-Herzegovina. All participants were approached and recruited through their respective diabetes type 1 patient organizations in Bugojno and Vitez. The network utilized a mobile health app and remote digital monitoring to bridge the gap between the main pediatric diabetology clinic in Sarajevo and the remote regions of Middle Bosnia Canton. The network also included local pediatricians and doctors from the hospital in Bugojno, Middle Bosnia Canton, Additionally, an online Viber peer support chat group was integrated into the network, facilitating daily communication and exchange among patients and healthcare providers. The establishment and complete structure of the digital health network has been described elsewhere (47).

Patients with T1DM were divided into two age groups, children aged ≤12 years, along with their parents, and adolescents/young adults aged >12 years. This division was based on the rationale that parents of children aged ≤12 years are generally responsible for managing T1DM in their children, whereas adolescents/young adults during the period of transition of care generally require less support from their parents and start managing their disease on their own (40, 42). Three online versions of the survey (i.e., for children/parents, adolescents/young adults, and HCPs) were created using Qualtrics XM®. The links, which were generated separately, were distributed through email and WhatsApp groups to the enrolled T1DM pediatric patients/parents and HCPs. Data were collected within 3 months after the end of the mHealth-based T1DM management program to minimize recall bias. The questionnaires were kept short to reduce respondent fatigue.

### 2.2 mHealth app intervention

**Figure 1:**
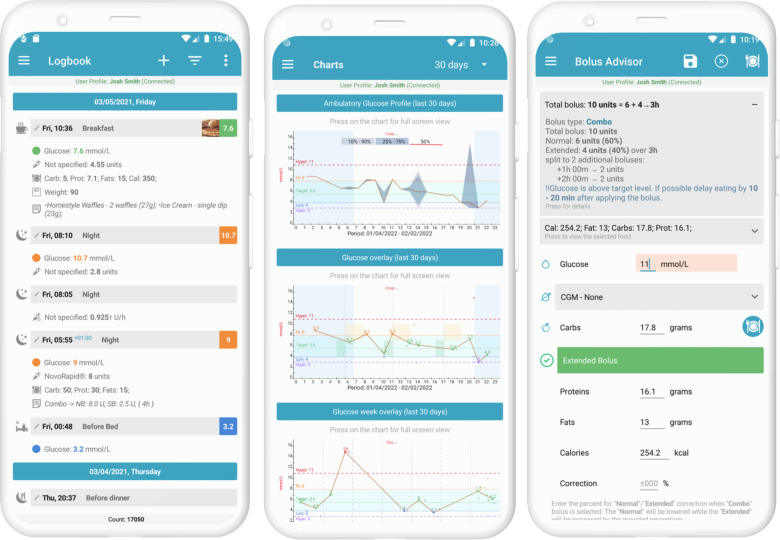
Screenshots of diabetes: M app key features. Photo Credit (https://diabetes-m.com/features/).

**Figure 2:**
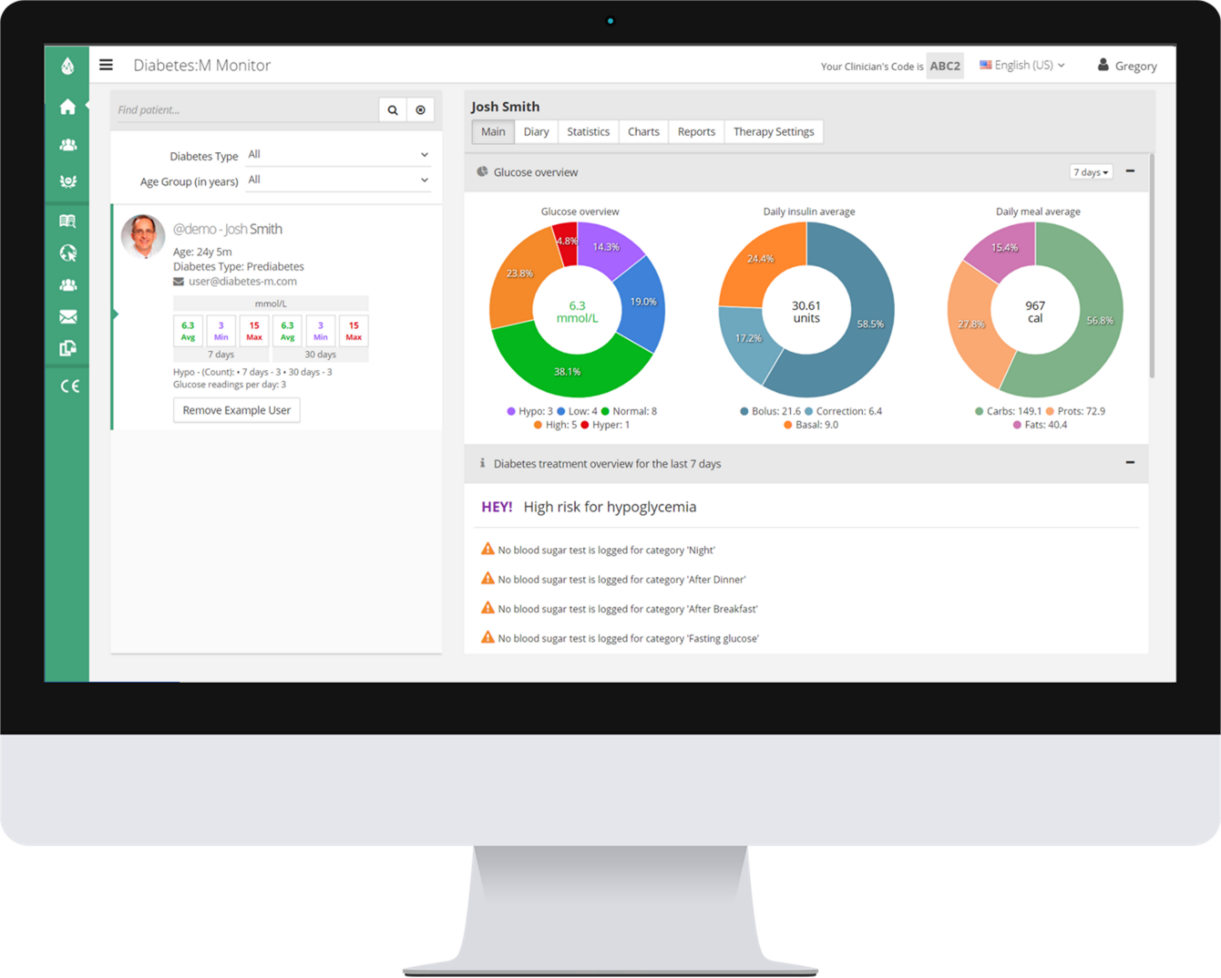
Diabetes: M remote monitoring tool for professionals. Photo Credit (https://diabetes-m.com/monitor/).

**Figure 3:**
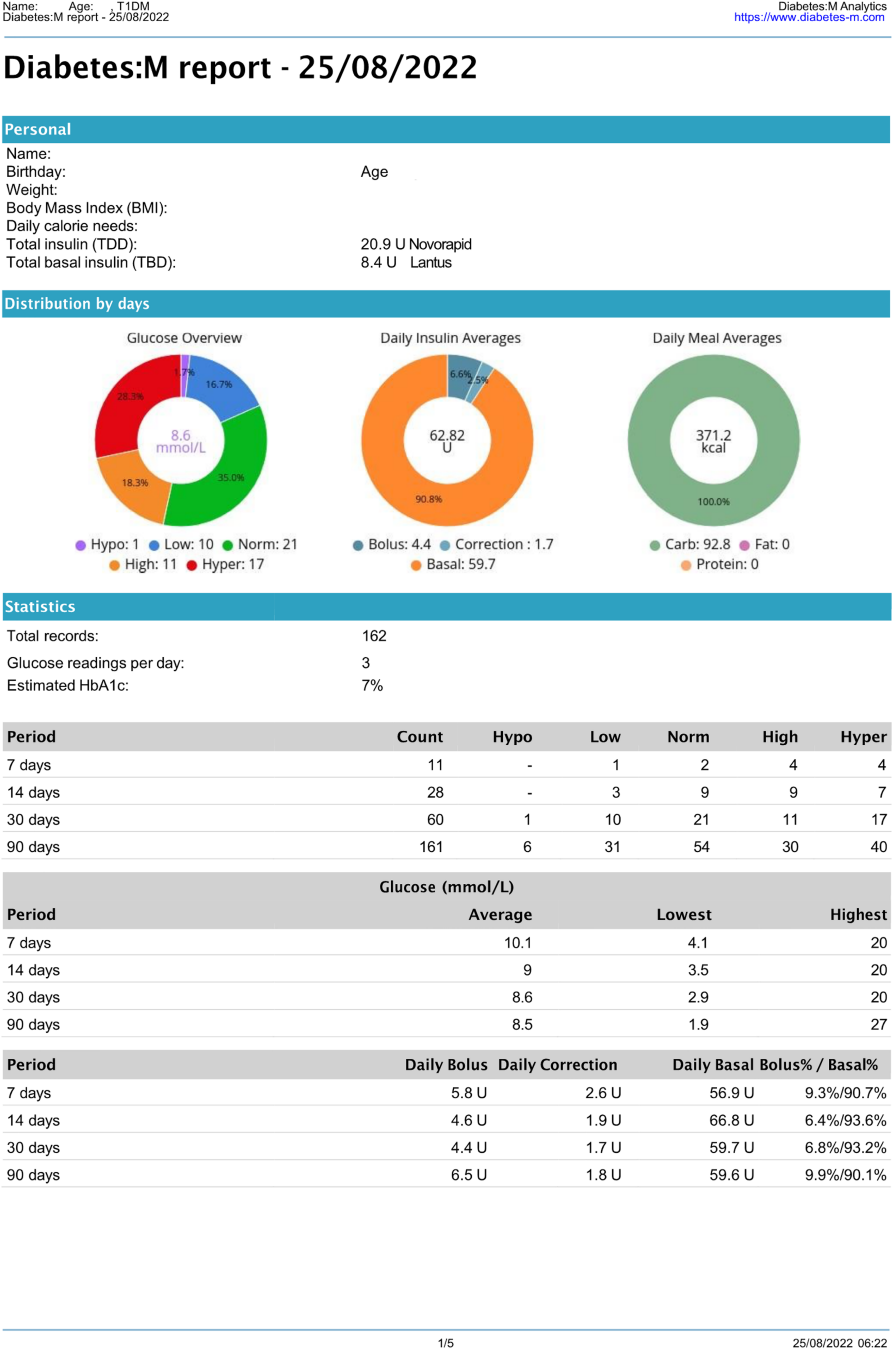
Anonymous 14-year-old T1DM patient's Diabetes: M report. (1st page only).

The Diabetes: M app platform consists of a mobile app (Figure 1) and a clinician monitoring system (Figures 2, 3) developed by Sirma Medical Systems. This app has been shown useful in improving diabetes control in T1DM patients (48) and is used in managing and monitoring all types of patients with diabetes and pre-diabetes. Table 1 shows a detailed description of its key features.

**Table 1:** Key features of the diabetes: M app, an interactive mHealth app consisting of a smartphone application for patients and a remote monitor software platform for HCPs.

Functions	Description
Food database	The food database provides a categorized list of the most common foods and products. It also integrates with external food databases and allows customized entries of products and portions.
Logbook	The logbook function allows the entry of glucose concentrations, insulin injections, and carbohydrate amounts. Additional values, including ketones, cholesterol, HbA1c levels, weight, blood pressure, pulse, and physical activities, can also be added.
Bolus calculator	The bolus calculator calculates insulin units based on carbohydrate, protein, and fat intake or on measured glucose concentrations and notifies about the need for additional carbohydrates or to delay meals due to high blood glucose concentrations.
Reminders	The reminder function reminds user of various tasks, such as taking medications and scheduling doctor appointments.
Charts and graphs	Entries including blood sugar concentrations, medications, and physical activities can be viewed as graphs over time (daily, weekly, monthly, and yearly). This application also includes analytical charts with more detailed and various types of data.
Pattern analysis	The pattern recognition function allows analyses of data over the previous 14 days and helps recognize issues associated with behavior and adherence to treatment. This app also promotes increased understanding of insulin dosing, correction effects, and glycemic variability.
Reports	The report function can generate very detailed reports in various formats (i.e., pdf, XLS, and HTML) and can share them with HCPs.
Online monitoring and follow-up by HCPs^a^	The remote monitoring tool provides HCPs with real-time patient information. This tool promotes monitoring, coaching, and education of patients in close connection with their HCPs.

^a^ HCPs, healthcare professionals.

### 2.3 Outcome measures

The three questionnaires, for T1DM children/parents, adolescents/young adults, and HCPs, were each divided into four main parts: participant characteristics, satisfaction and ease of use, the usefulness of different app features, and user experience with the app in managing T1DM. The questionnaires were developed in English, then translated into Bosnian by forward and back translation methods and adapted for cultural considerations. The two experts in pediatric diabetology checked these questionnaires for face and content validity. Based on their feedback, each questionnaire was revised to its final form. Participant information sheets were also evaluated for comprehensibility and appropriateness.

#### 2.3.1 Participant characteristics

The first part of the questionnaires included questions about the sociodemographic characteristics of the participants, including age, gender, previous experience with mHealth apps, professional experience, and healthcare specialty (in the HCP questionnaire only).

#### 2.3.2 Satisfaction and ease of use

The primary objective of the usability element was to assess patient and provider satisfaction and ease of use of the app. This objective was measured using the 8-item subscale of the Mobile Health App Usability Questionnaire (MAUQ) (49), a reliable and validated questionnaire with four different versions depending on the type of app (interactive or standalone) and the target user of the app (patient or provider). The MAUQ Interactive app, provider, and patient versions were selected for this study. Satisfaction and ease of use were measured using a 7-point Likert-type scale, ranging from 1 (strongly disagree) to 7 (strongly agree). A higher score indicated greater satisfaction with and ease of use of the app.

#### 2.3.3 Usefulness of different app features

Assessments of the app usability included evaluations of the usefulness of different app features, namely logbook, food database, reminders, bolus calculator, charts and graphs, reports, pattern analysis, and online monitoring by HCPs (8 items). Each item was graded on a 5-point Likert-type scale, ranging from 1 (not at all useful) to 5 (extremely useful). A higher score indicated greater perceived usefulness of the app feature. Participants were also provided with the option “I have not used this feature.”

#### 2.3.4 User experience with the app

The fourth part of the questionnaires consisted of questions examining participants' experiences with the app in the management of T1DM. These questions, based on previous studies (24, 49, 50), were adjusted depending on the populations being assessed i.e., pediatric patients, parents, and providers. All of these questions were dichotomous, although participants could also state that they were uncertain.

### 2.4 Data analysis

Data were extracted from QualtricsXM® and descriptive statistics were analyzed using Microsoft Excel (2019). Categorical variables were reported as frequencies and percentages, whereas continuous variables were reported as means, standard deviations (SD), and ranges.

### 2.5 Ethical considerations

The current study is part of the project “Improving Diabetes Type 1 Care in Children and Adolescents in Bosnia and Herzegovina” (51) and was approved by the Ethics Committee of the Medical Association of the Central Bosnian Canton (Ethical Approval number 839/22). Subjects were provided with a detailed participant information sheet and informed consent/assent documents, with all participants providing written informed consent/assent using the checkbox option before starting the online survey. The individual questions were not linked and could be skipped to continue the questionnaire. The anonymity of the participants was ensured at all times, and the study was conducted in accordance with the Declaration of Helsinki (52).

## 3 Results

### 3.1 Outcome measures

#### 3.1.1 Participant characteristics

From August to September 2022, 50 children, adolescents, and young adults diagnosed with T1DM were screened and enrolled in a 3-month mHealth-based T1DM management program. In addition, nine HCPs were trained in the use of the mHealth app in diabetes management as part of the digital health network (47). All 50 T1DM patients (children, adolescents, and young adults) and all nine HCPs completed the program and responded to the online survey, leading to response rates of 100% The mean ± SD age of T1DM patients was 14 ± 4.54 years (Table 2). Thirty-five (70%) were aged >12 years, 26 (52%) were female, and 37 (74%) reported previous experience with mHealth apps. The mean ± SD age of the HCPs was 43.4 ± 7.76 years; all nine (100%) were women and had a mean ± SD professional experience of 17.8 ± 8.81 years. Five (56%) of the nine HCPs had no previous experience using mHealth apps professionally. The nine HCPs included seven physicians, one pharmacist, and one other healthcare professional.

**Table 2:** Demographic characteristics of the participants included in this cross-sectional study.

Characteristics	Values
Characteristics of T1DM children/adolescents (*N* = 50)^a^	
Age (years, mean ± SD)	14 ± 4.54
Age categories, *n* (%)	
≤12 years	15 (30)
>12 years	35 (70)
Gender, *n* (%)	
Male	24 (48)
Female	26 (52)
Previous experience with mHealth apps, *n* (%)	
Yes	37 (74)
No	13 (26)
Characteristics of healthcare professionals (*N* = 9)	
Age (years, mean ± SD)	43.4 ± 7.76
Gender, *n* (%)	
Male	0 (0)
Female	9 (100)
Professional experience (years, mean ± SD)	17.8 ± 8.81
Previous professional experience with mHealth apps, *n* (%)	
Yes	4 (44.4)
No	5 (55.6)
Health care specialty, *n* (%)	
Physician	7 (77.8)
Pharmacist	1 (11.1)
Nurse	0 (0)
Others	1 (11.1)

^a^ Parents filled out the questionnaires for children aged ≤12 years.

#### 3.1.2 Satisfaction and ease of use

**Figure 4:**
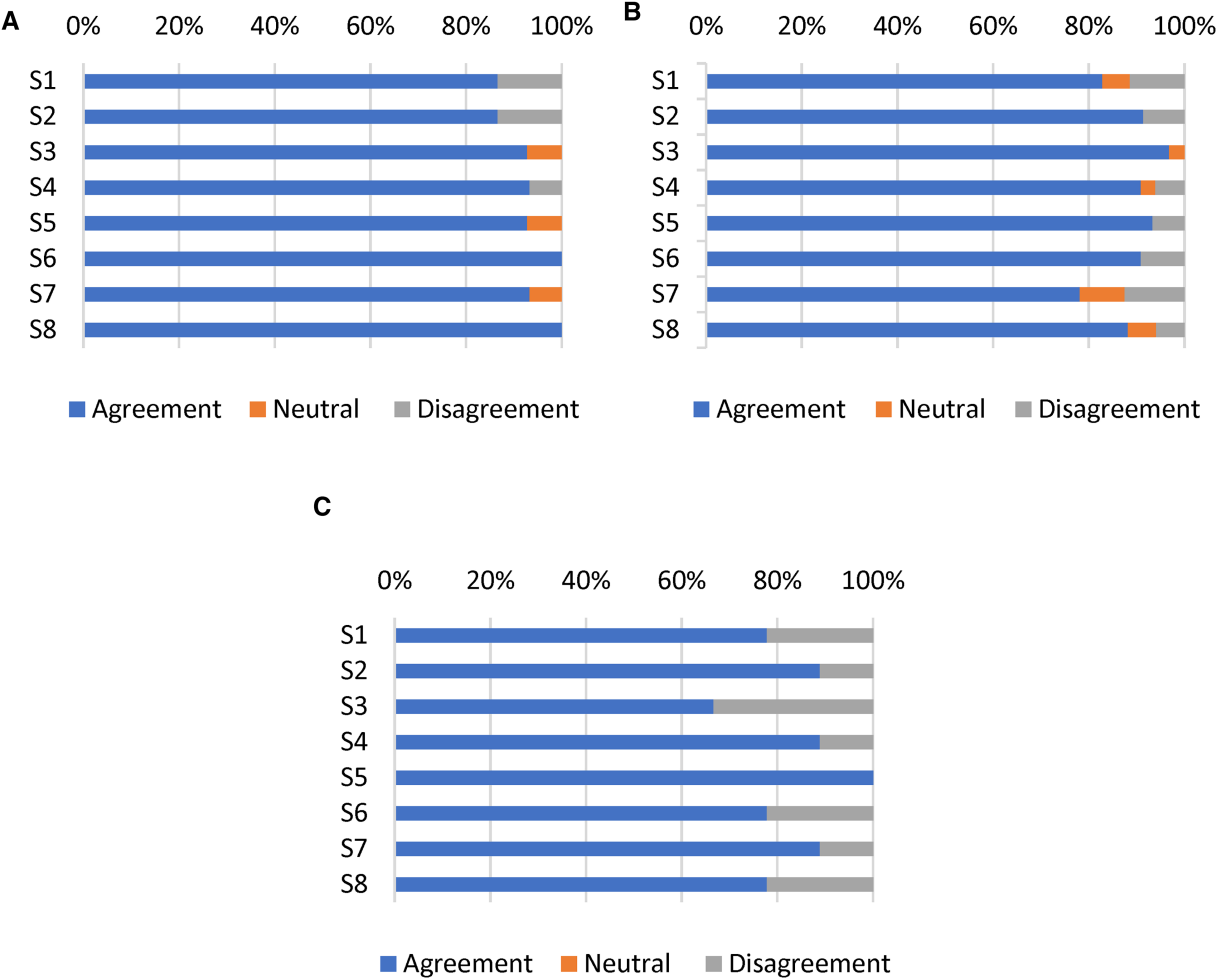
Responses of (**A**) T1DM children/parents, (**B**) T1DM adolescents/young adults, and (**C**) HCPs to eight items on the satisfaction and ease of use subscale of the MAUQ. Results reported as the percentages of participants who achieved scores on a 7-point Likert scale (Strongly agree, agree, and somewhat agree defined as agreement; strongly disagree, disagree, and somewhat disagree defined as disagreement).

Satisfaction with the mHealth app intervention was determined by measuring eight items on the MAUQ subscale for satisfaction and ease of use. The app was reported usable in the domains of ease-of-use and satisfaction by the T1DM children/parents (5.82/7.0), T1DM adolescents/young adults (5.68/7.0), and HCPs (5.22/7.0), indicating that all participants rated the Diabetes: M app as satisfactory (Table 3). The agreement among T1DM pediatric patients/parents for satisfaction and ease of use was >80% (Figures 4A, 4B), whereas the agreement among HCPs was 74.5% (Figure 4C).

**Table 3:** Satisfaction of the participants with the diabetes: M app, measured using the mHealth App usability questionnaire (MAUQ) sub-scale for satisfaction and ease of use.

Variables (scored on a scale of 1–7)^a^	T1DM Children/parents Mean (SD)	Range	T1DM Adolescents/young adults Mean (SD)	Range	Healthcare professionals Mean (SD)	Range
S1-The app was easy to use.	5.33 (1.84)	1–7	5.63 (1.42)	1–7	4.88 (2.32)	1–7
S2-It was easy for me to learn to use the app.	5.67 (1.63)	1–7	5.60 (1.56)	1–7	5.56 (1.81)	1–7
S3-I like the interface of the app.	5.93 (0.83)	4–7	6.06 (0.76)	4–7	4.44 (2.60)	1–7
S4-The information in the app was well organized, so I could easily find the information I needed.	5.80 (0.86)	3–7	5.52 (1.18)	1–7	5.44 (1.81)	1–7
S5-I feel comfortable using this app in social settings.	5.79 (0.80)	4–7	5.87 (1.36)	1–7	6.13 (0.4)	6–7
S6-The amount of time involved in using this app has been fitting for me.	5.93 (0.59)	5–7	5.64 (1.29)	2–7	5.11 (1.9)	1–7
S7-I would use this app again.	5.93 (0.80)	4–7	5.41 (1.60)	1–7	5.44 (1.81)	1–7
S8-Overall, I am satisfied with this app.	6.20 (0.56)	5–7	5.71 (1.38)	1–7	5.11 (1.9)	1–7
All satisfaction items	5.82 (0.25)	1–7	5.68 (0.21)	1–7	5.22 (0.38)	1–7

^a^ Response categories: 1 = strongly disagree, 2 = disagree, 3 = somewhat disagree, 4 = neither agree nor disagree, 5 = somewhat agree, 6 = agree, 7 = strongly agree.

#### 3.1.3 Usefulness of app features

**Figure 5:**
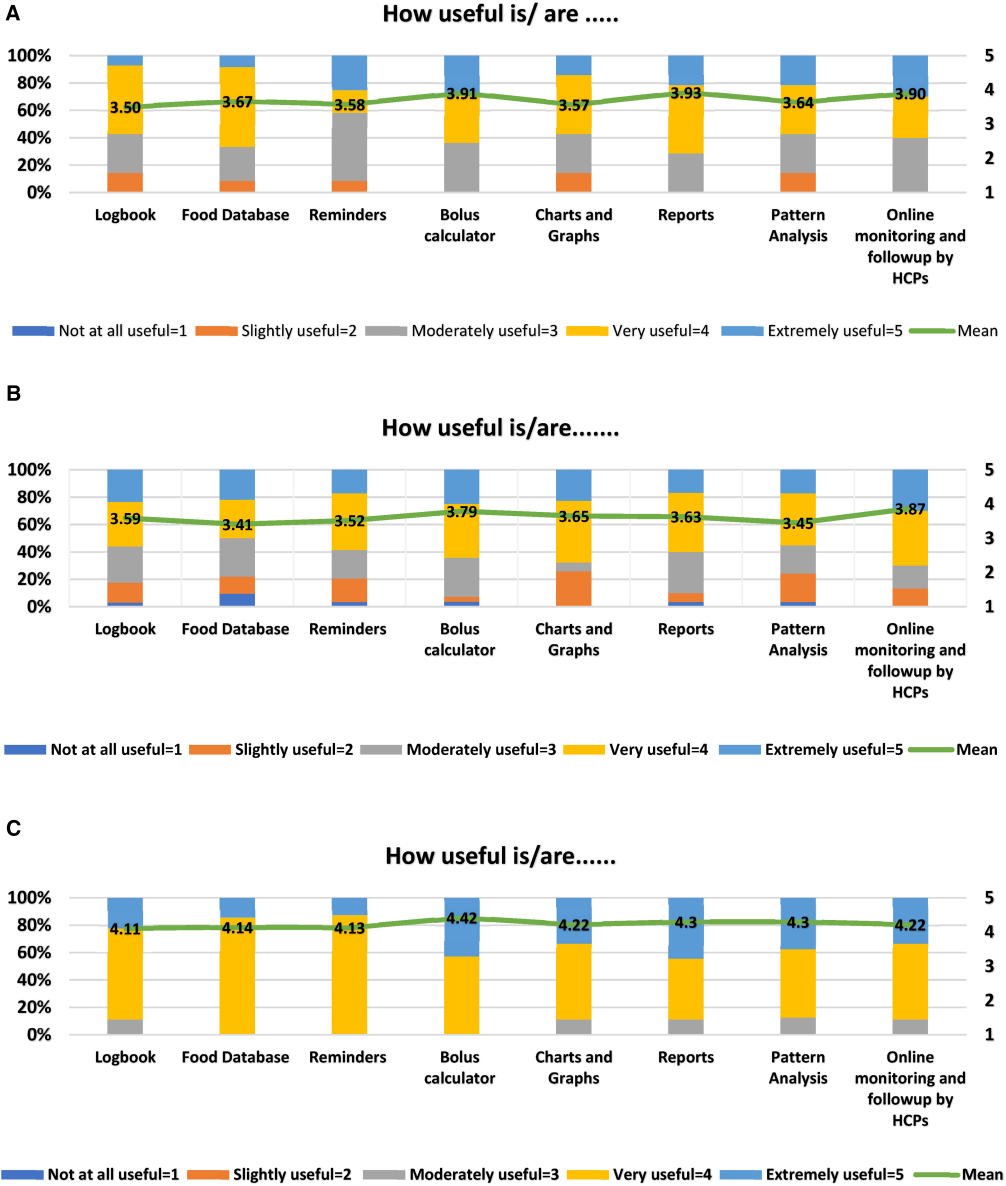
(**A**) diabetes: M app features rated by children/parents (*n* = 15). Mean values are shown in the bars*.* (**B**) Diabetes: M app features rated by adolescents/young adults (*n* = 35). Mean values are shown in the bars. (**C**) Diabetes: M app features rated by HCPs (*n* = 09). Mean values are shown in the bars.

The mean usefulness score was more than 3 for all 8 features in both patient age groups. The top three key app features rated by T1DM children/parents who used these features during the 3-month trial of the T1DM management program were the reports (3.93/5), bolus calculator (3.91/5), and online monitoring and follow-up by HCPs (3.90/5) (Figure 5A). Similarly, T1DM adolescents and young adults found online monitoring and follow-up by HCPs (3.87/5), bolus calculator (3.79/5), and charts and graphs (3.65/5) to be more useful (Figure 5B). The HCPs rated bolus calculator (4.42/5), pattern analysis (4.3/5), and reports (4.3/5) to be the top three “extremely useful/very useful” app features for T1DM management with a mean usefulness score of more than 4 for all 8 features (Figure 5C).

#### 3.1.4 User experience with the app

The nine HCPs rated their experiences with the Diabetes: M app in managing and supporting T1DM care of their pediatric patients as highly favorable. Only two of the nine HCPs encountered technical issues, with six reporting improvements in managing their patients with diabetes. Eight of the HCPs reported that use of the app improved their communications with patients, their understanding of patients' diabetes management, and their sharing of information and other educational material (Table 4).

**Table 4:** HCPs' experience with the app (*N* = 9).

Statement	Yes %
Have you noticed an improvement in your patient's diabetes management while using the app?	67
Were there any technical issues or bugs you encountered while using the app?	22
Did the app promote communication with your patients and their parents?	89
Did the app help you better understand your patient's type 1 diabetes management?	89
Were you able to send information and other educational materials to your patients and their parents through the app?	89

Of the 15 parents evaluated, seven (47%) noticed improvements in their child's T1DM management while using the app. Only four (27%) noticed changes in their child's daily activities, with two (13%) encountering technical problems while using the app. Eight (54%) parents agreed that the app helped them communicate with HCPs, and ten (67%) found that use of the app provided better understanding of their child's T1DM management (Table 5).

**Table 5:** Parents’ experience with the app (*N* = 15).

Statement	Yes %
Have you noticed any improvements in your child's diabetes management since using the app?	47
Did you find any changes in your child's daily activities?	27
Were there any technical issues or bugs you encountered while using the app?	13
Did the app help you communicate with your child's healthcare provider?	53
Did the app help you better understand your child's type 1 diabetes management?	67

Of the 35 adolescents/young adults, 15 (43%) noticed improvements in their diabetes management, whereas only six (17%) encountered technical issues while using the app. Sixteen (46%) of the 35 adolescents/young adults agreed that the app helped them communicate better with their HCPs. Nineteen (54%) adolescents/young adults found that the app enabled better management of their T1DM while traveling or during unexpected events, with Twenty-two (63%) being better able to understand their diabetes management (Table 6).

**Table 6:** Adolescents'/young adults' experience with the app (*N* = 35).

Statement	Yes %
Have you noticed any improvements in your diabetes management since using the app?	43
Were there any technical issues or bugs you encountered while using the app?	17
Did the app help you communicate with your healthcare provider?	46
Did the app help you manage your diabetes while traveling or during unexpected events?	54
Did the app help you better understand managing type 1 diabetes?	63

## 4 Discussion

This study provides insights into the initial usability of the mHealth app intervention among T1DM pediatric patients/parents and their HCPs. Our findings provide clues to the management of T1DM pediatric patients with limited access to healthcare facilities, especially to patients living in rural and remote areas. The survey items focused on the subjective evaluations by T1DM patients and their parents and by HCPs of satisfaction, ease of use, usefulness, and experience with the mHealth app intervention following the completion of a 3-month mHealth-based T1DM management program. At the end of the 3-month trial, the participants expressed satisfaction with the Diabetes: M app, with children/parents having an overall mean ± SD satisfaction score of 5.82 ± 0.25, adolescents/young adults with an overall mean ± SD satisfaction score of 5.68 ± 0.21, and HCPs having an overall mean ± SD satisfaction score of 5.22 ± 0.38, each on a 7-point Likert-type scale. Moreover, HCPs had a largely positive view of the usefulness of different app features and rated the app as more efficient than T1DM pediatric patients and their parents.

Patient satisfaction, defined as outcomes meeting patient expectations (53), has been associated with greater compliance to devised treatment plans and more improved outcomes (54). Satisfaction with patient-centered mHealth technologies is usually assessed using descriptive and quantitative measurements (53). The high mean overall satisfaction scores among T1DM pediatric patients and their parents in the present study indicate high satisfaction and ease of use in engaging with the mHealth app. This is in line with previous studies showing high levels of satisfaction with mHealth apps among children and adolescents with T1DM, as well as their parents (24, 42, 43, 55–58).

As revealed by different items on the MAUQ multi-item sub-scale (49), T1DM pediatric patients and their parents reported that the app was easy to use and easy to learn to use. These participants liked the app interface and found that the information component of the app was well-organized and easily accessible. They felt comfortable using the app in social settings, similar to findings showing that adolescents found that mHealth apps were an acceptable method of communicating with their parents, particularly in social settings (42). Participants' satisfaction in the present study was also shown by their intention to use the app again, similar to previous results showing that 96% of study subjects indicated intent to use the app if available outside the trial (43). “The amount of time involved in using this app has been fitting for me” was also rated positively by the respondents, as was the overall satisfaction with the app. These high scores for satisfaction and ease of use may have been due to the ease of use of the simple platform and the familiarity of younger patients with mHealth apps, as 74% reported previous use of mHealth apps. High patient satisfaction in our study may also be due to the prompt responses of HCPs to their patients via an online platform created specifically for this purpose. Moreover, the nine HCPs who took part in the study also rated the app as satisfactory. In other studies, HCPs have also reported high satisfaction with mHealth-based systems for chronic disease management in children and adolescents (24, 59).

Participants were also asked to rate the usefulness of different app features in assisting with daily T1DM self-management. Overall, pediatric patients and their parents found various app features to be “moderately useful to very useful,” whereas HCPs rated these app features to be “very useful to extremely useful.” Online monitoring and follow-up by HCPs, bolus calculator, and reports were the top three rated features by the T1DM children/parents. Similarly, adolescents and young adults found online monitoring and follow-up by HCPs, bolus calculator, and charts and graphs to be more useful. This contrasts with previous studies conducted in New Zealand and Canada where the logbook feature was among the most favored and used features (60, 61). We found clues for this finding in the local context of Bosnia and Herzegovina where the Diabetes: M app could not be integrated with continuous glucose monitoring (CGM) systems and the patients had to enter their blood glucose levels manually every time. This may have led to possible patient fatigue and less interest in blood glucose tracking feature and consequent usability. However, similar to our study a Scottish survey reported the blood glucose data feature was felt useful by a minority of patients and the bolus calculator was the most desired feature by T1DM patients (62). Connected through the digital health network, the patients were regularly monitored by the HCPs which is depicted by the favorable rating of online monitoring and follow-up feature by the patients and their parents. During the 3 month T1DM management program, the HCPs responded to immediate patient needs. Nevertheless, the frequency of remote monitoring and communication consistency can be decided mutually among HCPs and patients/parents that fit their daily activities. Moreover, scaling up such mHealth interventions requires an adequate number of dedicated well-trained HCPs, an appropriate workload, task shifting, and other encouragement approaches such as monetary incentives and opportunities for training (63–66). In our study, HCPs rated the bolus calculator, reports, and pattern analysis as the most highly useful features. Graphically and statistically generated reports and pattern analysis were rated as helpful by HCPs in another study of children and adolescents with T1DM (24).

Although HCPs were very positive about their experience with the Diabetes: M app in managing T1DM, pediatric patients and their parents rated their experience as moderate. For further implementation, it is therefore important to highlight all stakeholders' lived initial user experiences and insights. These findings about the optimal functional experience are also important for the long-term engagement of users and sustained use of apps beyond the initial adoption stage owing to the problem of high dropout rates and less user retention (67).

Although the results of the present study correspond with those of earlier studies, the present findings indicate that the mHealth solutions might fill the care gaps and compensate for a lack of functional health infrastructure in remote and rural areas with limited to almost no facilities. Patients in rural areas with limited healthcare access can get an advantage from digitally assisted remote care options, however, existing care should be supplemented gradually instead of substitution, keeping in mind the local context and individual patient characteristics (68). The favorable attitude of HCPs and T1DM pediatric patients/parents towards the mHealth app underlines their great interest and has led to increased adoption of mHealth care services by these populations (69, 70). Increased satisfaction with diabetes apps provides evidence for the increased implementation of mHealth interventions for the management of chronic diseases. Launching the diabetes program in BiH was not easy due to complexities in the political and economic situation in BiH and large regional disparities in diabetes care of children and adolescents. Unless they live in larger cities with pediatric diabetology clinics, these children do not have regular access to pediatric diabetes clinics or pediatric diabetologists. Children and their families often have to self-manage this complex and life-long disease, with late complications becoming inevitable. One of the key successes of this initiative was the active participation of patients and providers, with response rates of 100%. Moreover, this initiative included active parent involvement, improving T1DM management among children (71, 72), particularly through the mHealth apps (73). After enrollment into the digital health network, participants received formal training on how to use the mHealth app based on the significance of adequate training for using and implementing mHealth interventions (46, 74). This digital network can serve as an example for other remote or rural regions and it can accommodate other chronic disease management apps.

The present study had several limitations, including its use of self-reported measures, with these data subject to recall bias, possibly skewing the results. Another limitation was the small sample size, however, we had a minimal proportion of missing data. A third limitation was the short period of mHealth app usage of only 3 months. Finally, The current study may contain a response bias as all the HCPs, and T1DM patients/their parents voluntarily participated in the T1DM management program and subsequent survey, and might therefore be more enthusiastic and positive about the mHealth app or more motivated to use the app. Studies that include a larger number of patients and a longer period of mHealth-based T1DM management are needed to provide more robust conclusions.

## 5 Conclusion

A mobile app intervention, involving communications between pediatric patients with T1DM living in remote and rural areas of BiH and large specialized pediatric diabetology clinics in the capital city or other cities can facilitate diabetes care for the former. This app and other mHealth apps can be useful in overcoming the limitations of diabetes care in rural areas and improving diabetes management and HCP-patient communications. The positive experiences and satisfaction with the app reported by patients, their parents, and HCPs may be useful for other HCPs and policymakers in BiH and other countries with similar circumstances, suggesting that mHealth apps can facilitate the delivery of healthcare services. Randomized controlled trials with objective clinical outcomes, such as HbA1c and other glucose biomarkers, are needed to determine the efficacy and sustainability of mHealth interventions in pediatric patients with T1DM.

## Data Availability

The raw data supporting the conclusions of this article will be made available by the authors, without undue reservation.
